# Supplementary material for: Direct visualization of hydrogen absorption dynamics in individual palladium nanoparticles
Source: Nat Commun. 2017 Jan 16;8:14020. doi: 10.1038/ncomms14020 (PMC5241819; doi:10.1038/ncomms14020)
Supplement: Supplementary Information — Supplementary Figures, Supplementary Methods, Supplementary Discussion and Supplementary References [file ncomms14020-s1.pdf]

Supplementary Figures

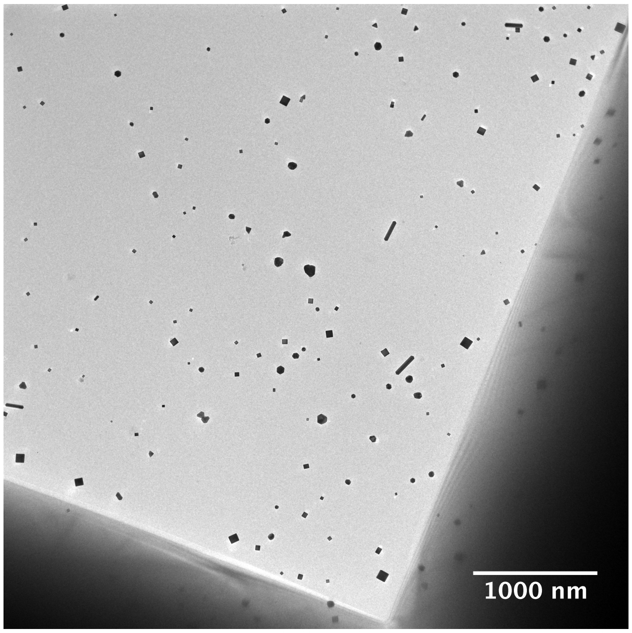

Supplementary Figure 1: **Representative sample**. Transmission electron microscopy image of a representative sample used in this study.

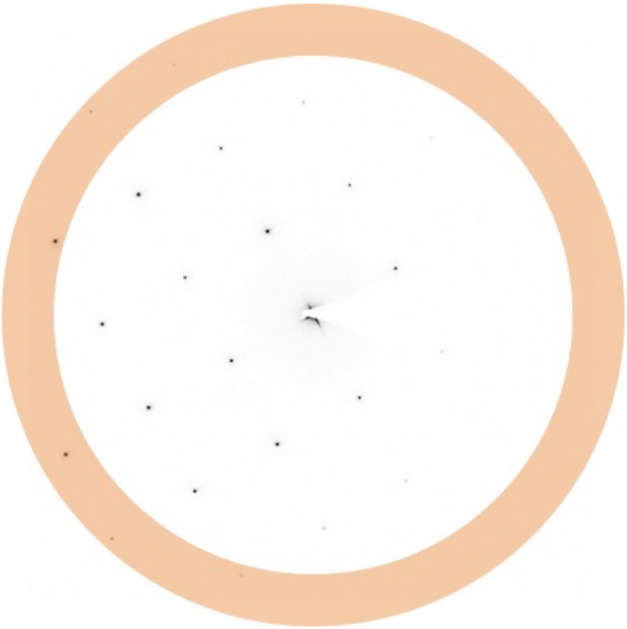

Supplementary Figure 2: **Origin of diffraction contrast in STEM**. Representative diffraction pattern of a nanocube. Shown in orange is the region in reciprocal space captured by the annular dark field detector. It can be seen that strongly diffracting spots are collected by the detector.

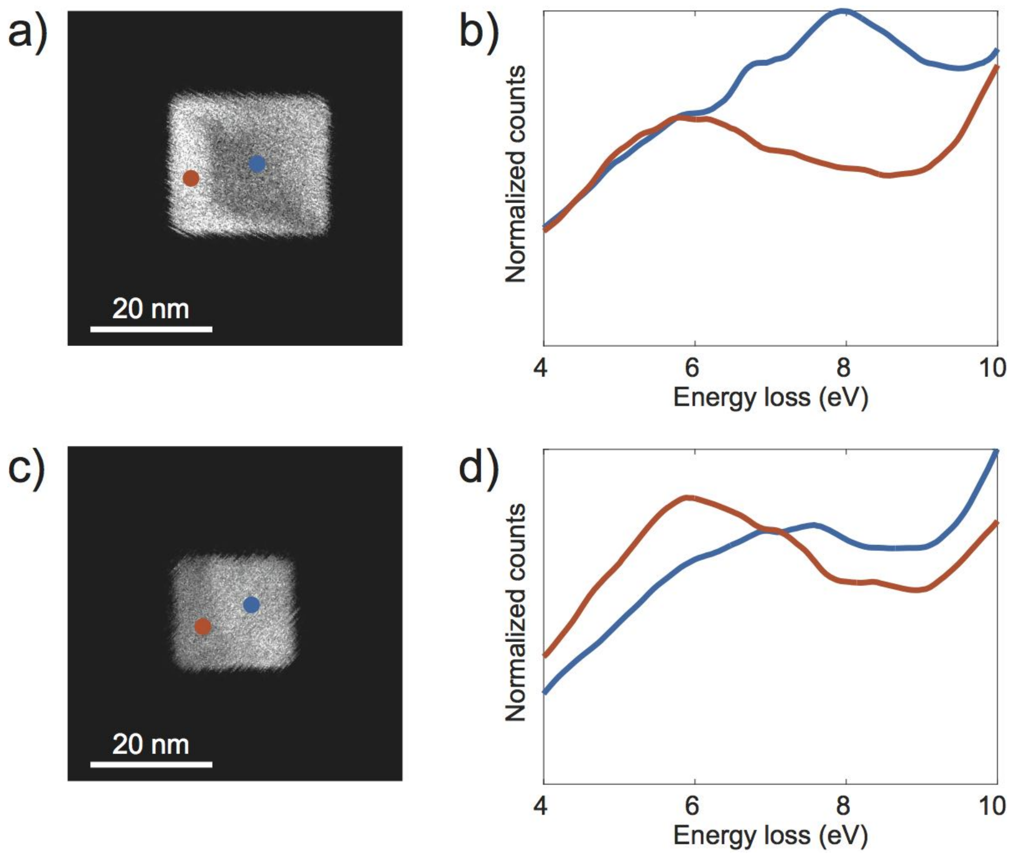

Supplementary Figure 3: **Correspondence of contrast differences with phase.** (a) and (c) Sample STEM images of particles showing phase coexistence. (b) and (d) Smoothed EEL spectra of the particles shown in (a) and (c) at the locations delineated by the blue and red dots. It is seen that the two particles shown have opposite contrast between the α and β phases.

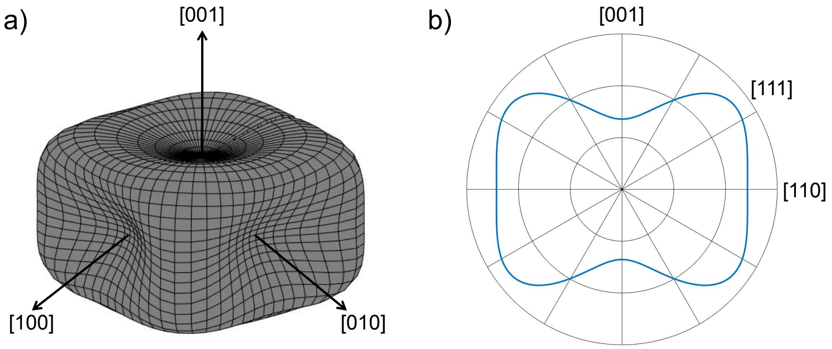

Supplementary Figure 4: **Orientation-dependent elastic energy penalty.** (a) Elastic energy penalty for a coherent boundary between the α and β phases of palladium hydride with an interface with normal **n**. The distance from the origin in a given direction corresponds to an energy penalty for a boundary with a normal along that direction. (b) A slice of the diagram shown in (a) highlighting behavior along low-index planes.

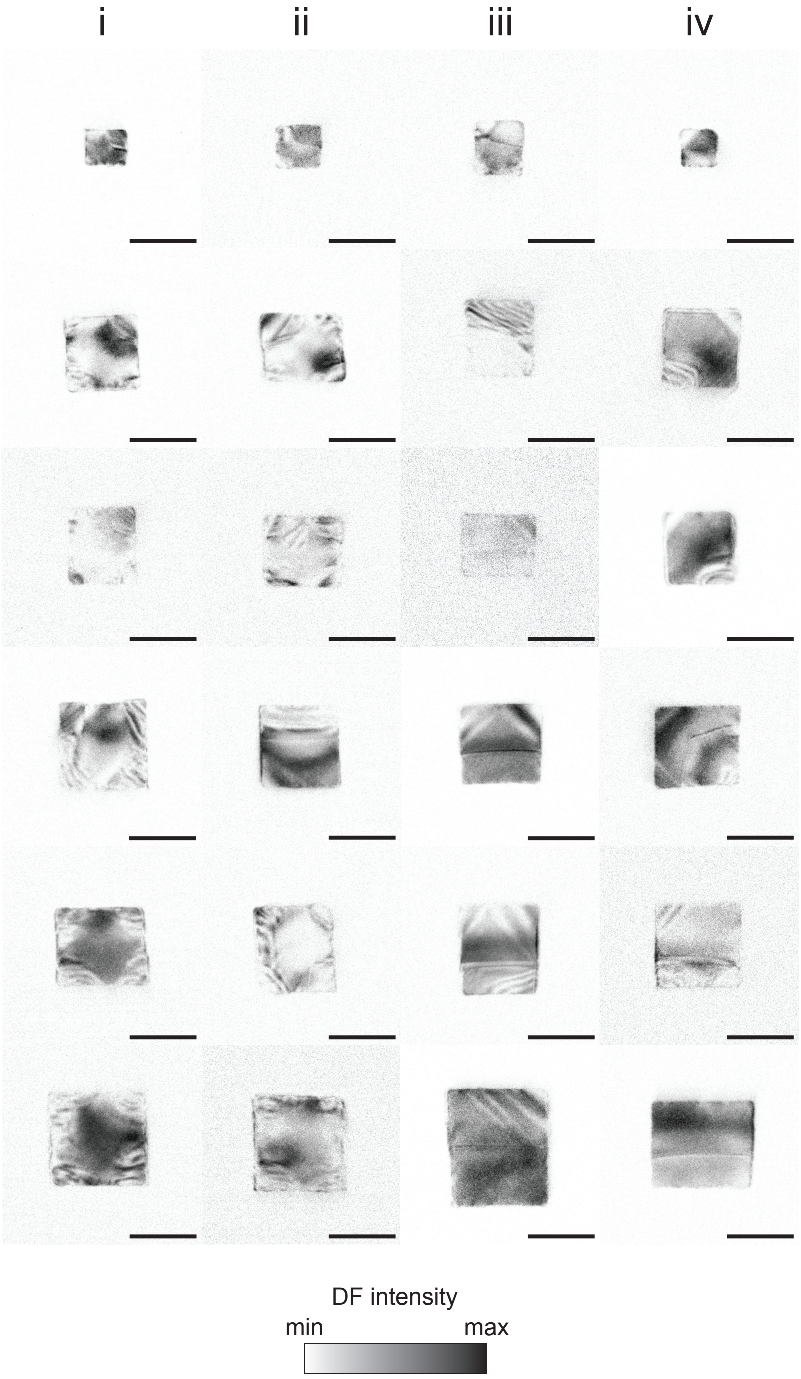

Supplementary Figure 5: **Entire-spot DF images.** Dark field images acquired by including both the α and β diffraction spots inside the objective aperture. The particles pictured here are those shown in Figure 2. Scale bars correspond to 50 nm.

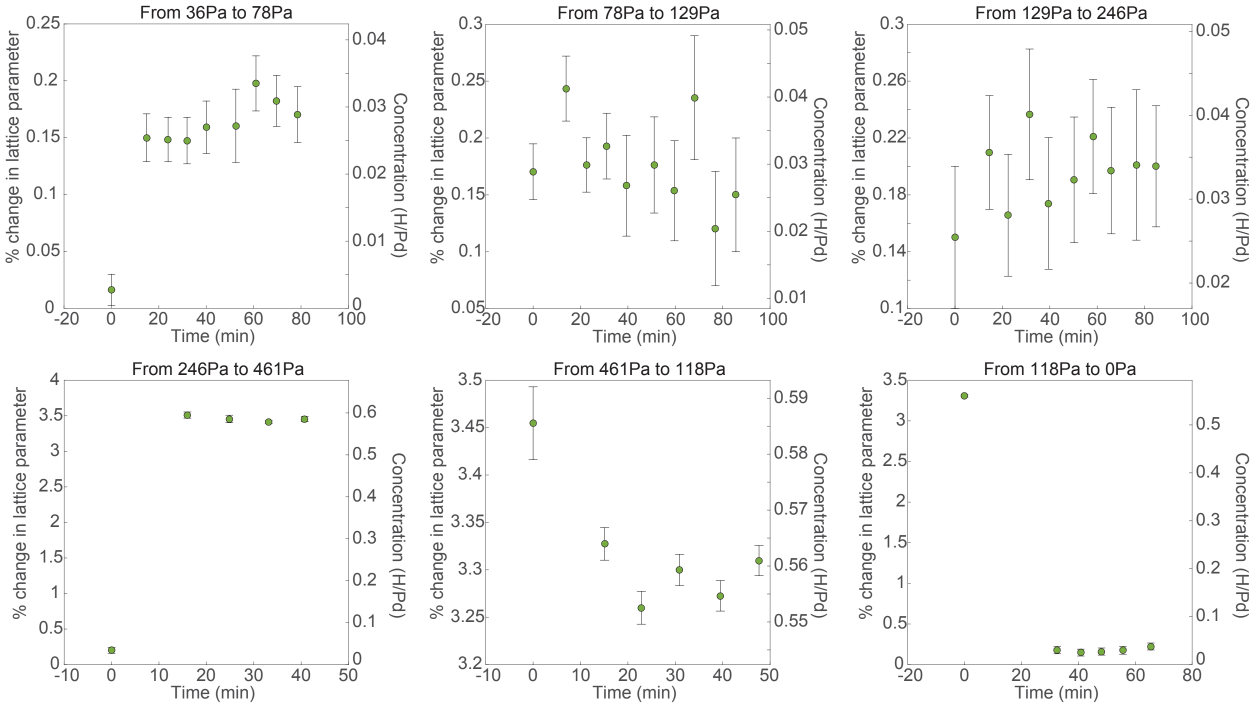

Supplementary Figure 6: **Equilibration of the nanocube**. A series of plots of the lattice parameter as a function of elapsed time after increasing the pressure by the amount specified over each plot. The data suggest that the system has equilibrated or at least reached a highly stable state within thirty minutes. The change in lattice parameter was determined by creating a distribution of the changes in spacing between all pairs of diffraction spots and comparing each spacing to that obtained before introducing hydrogen. The error bar corresponds to the standard deviation of that distribution.

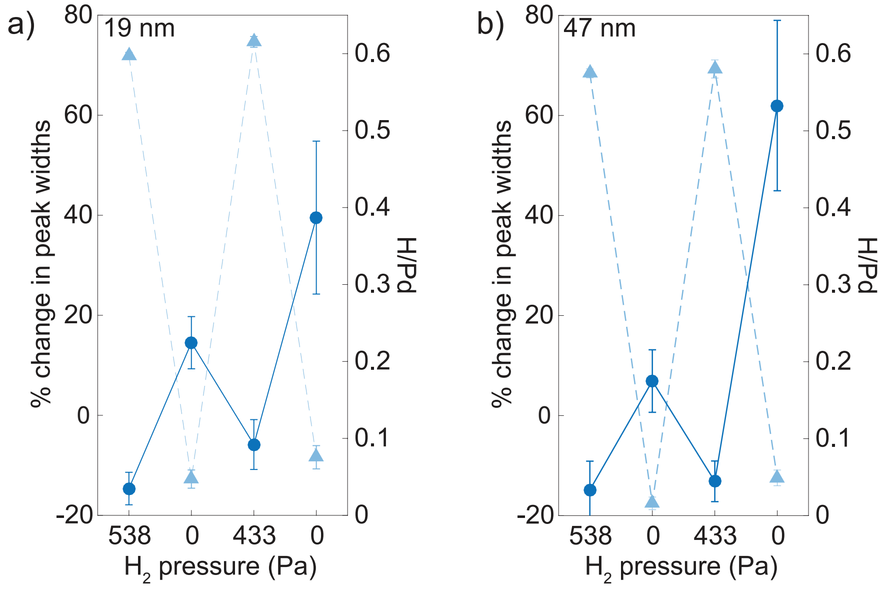

Supplementary Figure 7: **Impact of cycling on crystallinity in different sized particles.** Change in peak width upon cycling for (a) 19 nm and (b) 31 nm particles. The width of each spot is referenced to the original spot in the room temperature diffraction pattern. The standard deviation of this distribution of width changes corresponds to the error bar. The change in peak width is plotted using a circular point and the lattice parameter is plotted with a triangle. The trend seen in both particle sizes is very similar.

Supplementary methods

Sample preparation

Large nanocube synthesis

Pd nanocubes are synthesised by adapting a method that was previously reported[1]. In a typical synthesis, two separate batches of seeds are prepared. In two separate reaction vessels, 20 mL of a 12.5 mM and 6.25 mM aqueous CTAB solution is heated to 95°C while stirring in a 100 mL flask equipped with a condenser. 1 mL of a 10mM H_2PdCl_4 aqueous solution is then added and the mixture is stirred for 5 minutes. The H_2PdCl_4 solution is prepared by suspending 8.9 mg $PdCl_2$ (50.2 μmol) in 208 μL of 0.48 M HCl (100 μmol), diluting the suspension to 5 mL with water and then sonicating for approximately 15 minutes. 160 μL of 100 mM L-ascorbic acid aqueous solution is then added. The mixture is stirred at 95°C for 20 minutes and then cooled in a water bath. The CTAB-capped Pd nanocubes are washed twice by centrifugation in a 100 kDa filter centrifuge tube (4000xg, 14 minutes) and redispersed in millipore water. The reaction using 6.25 mM CTAB has not been reported before and yields seeds of 20 ± 4 nm. Larger nanocubes are synthesized by adapting a literature method for growth of the nanocube seeds[1, 2]. The larger nanocubes were 28 ± 2 nm, 32 ± 3 nm, 54 ± 6 nm, and 70 ± 18 nm. In a typical synthesis, four sets of nanoparticles are prepared in parallel; each synthesis was performed in a 15 mL centrifuge tube. Four sets of 5 mL of 50 mM CTAB were warmed to 60°C. The reaction vessels were removed from the water bath and three solutions were quickly added. 125 μ L of 10 mM H_2PdCl_4 was added to each followed by an appropriate amount of unwashed seed solution: 28 nm: 500 µL of 6.25 mM CTAB, 32 nm: 250 µL of 6.25 mM CTAB, 54 nm: 250 µL of 12.5 mM CTAB, 70 nm:80 µL of 12.5 mM CTAB. Finally, 50 μ L of 100 mM ascorbic acid was added to each. The resulting solution was gently mixed by hand and then placed back into the 60° water bath. The reaction was allowed to proceed for 2 hours.

The purification of the 28 and 32 nm cubes was as follows: 3 1 mL aliquots of the reaction mixture were centrifuged at 3500xg for 13 minutes. After removing the supernatant, 333 μ L of 1 μ M CTAB was added to each tube and the aliquots were combined and centrifuged at 3000xg for 13 minutes. The supernatant was removed, 1 mL of 1 μ M CTAB was added, and the particles were centriguged at 3000 xg for 13 minutes. The supernatant was removed and 750 μ L of 1 μ M CTAB was added.

The purification of the 54 and 70 nm cubes was as follows: 5 1 mL aliquots of the reaction mixture were centrifuged at 1500xg for 13 minutes. After removing the supernatant, 200 μ L of 1 μ M CTAB was added to each tube and the aliquots were combined and centrifuged at 1000xg for 13 minutes. The supernatant was removed, 1 mL of 1 μ M CTAB was added, and the particles were centriguged at 1000 xg for 13 minutes. The supernatant was removed and 500 μ L of 1 μ M CTAB was added.

TEM grid preparation

100 μ L of the two seed solutions and the four growth solutions were combined in a centrifuge tube. 10 μ L of the mixture is pipetted onto a 20 nm thick silicon dioxide grid (Structure Probe, Inc., SO100-A20Q33), which had previously been plasma cleaned for 1 minute in air plasma at 10W. After 7 minutes and 15 seconds, the remaining liquid is wicked off with a Kimwipe. A representative TEM image of a sample is shown in Supplementary Fig. 1.

Microscopy

The SiO_2 membrane with the Pd nanoparticles is cleaned for one minute in an Ar/O_2 plasma at 50 W of RF power and then mounted on a TEM cryo holder (Gatan, Inc.) that allows control of the temperature to $\pm$ 0.1 K. Our environmental TEM allows us to vary the H_2 pressure between 4 and $\sim$ 600 Pa using a home-built mass flow controller operated gas manifold system. In order to avoid condensation of contaminants on the sample during hydrogen gas flow, we use a liquid nitrogen cooled cold finger during our environmental TEM experiments, which minimises beam-induced hydrocarbon contamination during STEM-EELS and SAED data acquisition at all hydrogen pressures. All experiments are carried out using an FEI Titan 80-300 environmental (scanning) transmission electron microscope operated at 80 kV for the EELS experiments and 300 kV for high-resolution imaging and diffraction, as described later. The lower voltage allows for a higher interaction cross section for spectroscopy whereas the higher voltage allows for higher resolution imaging. The microscope is equipped with a monochromator, an aberration corrector in the image forming objective lens and a Gatan 966 Quantum electron energy loss spectrometer. The H_2 (99.9999%, Praxair) pressure in the microscope chamber is monitored using an Edwards Barocell 600 capacitance manometer with a precision of $\pm$4%.

Diffraction-based isotherms

The diffraction patterns were obtained at 300 kV using a 180 nm selected area aperture. The electron dose rate is 5 electrons/Å^2 s. Each diffraction pattern is acquired for 3 seconds. The diffraction patterns of 25 different particles are obtained at room temperature. The sample was then heated to 60°C in 400 Pa of hydrogen. After 30 minutes, the sample was slowly cooled to -35°C in 4 Pa of hydrogen. The pressure was then incremented to $\sim$ 600 Pa in 13 logarithmically-spaced steps and then reduced back to 4 Pa in 8 logarithmically-spaced steps. At each pressure, the system was allowed to reach a steady state for 30 minutes. After

that waiting period, diffraction patterns are obtained for each particle. The pressure is then incremented upwards and the process is repeated.

The positions of all available diffraction spots in the room temperature reference pattern are determined using a 2D voigt function (the product of a voigt function in the x and y directions that is allowed to rotate around the z axis). The positions of those spots in the series of pressure-dependent diffraction patterns were obtained by the same fitting methods. The separation distance between all possible pairs of spots is then recorded and then expressed as a percentage change from the spacing in the room temperature reference pattern. The reported percent change is an average of all of the individual changes in the spacings and the uncertainty is the standard error of the measured distribution.

In order to find the equilibration time, or at least the time required to reach a steady state, we incremented the pressure and then repeatedly measured the diffraction pattern of ten different cubes. The data from one representative cube is shown in Supplementary Fig. 6. It is apparent that the concentrations stablilize within 30 minutes.

STEM-EELS live reaction monitoring

The sample was first heated to 60°C in 300 Pa of hydrogen gas. After 30 minutes, the sample was cooled to −27°C in 50 Pa of hydrogen gas. The pressure was then increased to 283 Pa. After finding a particle of interest, the video recording was started. After rastering the electron beam across a particle of interest for a few seconds, it began to absorb hydrogen as evidenced by the appearance of a region of different contrast at one of the corners in STEM. At the voltage and camera length used in this experiment, the annular dark field detector collects angles from 55 to 70 mrad. For the α and β phases of palladium hydride, the detector will collect the 440-, 600-, and 620-type reflections, meaning that the resulting STEM image has diffraction contrast. The relative contrast between the α and β phases is variable, as neither face consistently has higher contrast as observed in Supplementary Fig. 3. Periodically, the STEM image is stopped and EEL spectra are collected at several

points across the particle to assign the phase that corresponds to each region. Each EEL spectra was acquired for 0.4 seconds. The recording is stopped once the reaction is complete.

Dark field imaging and electron diffraction of frozen-in states

All of the imaging and diffraction patterns in this experiment were obtained at 300 kV. At room temperature before hydrogen introduction, the electron diffraction patterns of several particles were taken at an intensity of 10 electrons/Å^2 s with a 1 s exposure time. The sample was then heated to 60°C in 60 Pa of hydrogen. After 5 minutes, the sample was slowly cooled to -35°C to assure that the temperature did not undershoot by more than 0.5°C. The pressure was then increased to 250 Pa in slow increments to avoid overshooting. The leak valve was then opened by 100 units so that the system reached 500 Pa after 1 minute and 45 seconds. 2 minutes and 15 seconds after the leak valve was incremented, the heater current of the holder was shut off. As the holder cooled, the hydrogen was slowly removed from the microscope. Eventually, the holder stabilized at 100 K. Diffraction patterns were acquired for the particles originally examined at room temperature. For those particles exhibiting coexistence as evidenced by the diffraction pattern, the beam intensity was increased to 85 electrons/Å^2 s and the most intense diffraction point is determined. At this point, dark field imaging was performed. In this technique, an objective aperture is introduced in the diffraction plane to select intensity that arises from a diffraction spot. The image is then reconstructed using the electrons diffracting to the selected spot. We acquire three images – one in which the 10 μ m aperture contains the both the outside and inside spots, one in which it contains the outside of the two spots, and one in which it contains the inside of the two spots.

Supplementary Discussion

Coherent boundary energy penalty

Following the approach adopted by Cogswell and Bazant, we sought to approximate the elastic energy penalty sustained in an infinite medium of a phase boundary between the α and β phases with normal $\mathbf{n}$[3]. The energy penalty $B(\mathbf{n})$ is given by

$$B(\mathbf{n}) = C_{ijkl}\epsilon^0_{ij}\epsilon^0_{kl} - n_i\sigma^0_{ij}\Omega_{jl}(\mathbf{n})\sigma^0_{lm}n_m \qquad (1)$$

where

$$\Omega^{-1}_{ij} = C_{ijkl}n_k n_l \qquad (2)$$

Here, C_{ijkl} is the elastic stiffness tensor, ϵ^0_{ij} is the mismatch between the two phases and thus a diagonal matrix with each diagonal term equal to about 3.7%, and $\sigma^0_{ij} = C_{ijkl}\epsilon^0_{kl}$. The resulting plot of $B(\mathbf{n})$ is shown in Supplementary Fig. 4a. The radial distance is proportional to B. A projection showing the three low-index planes is shown in Supplementary Fig. 4b. This analysis shows that $\langle 100 \rangle$ is the preferred phase boundary orientation in an infinite medium. Although the exact numbers might be inaccurate upon nanosizing, the general trends are likely to persist.

Diffraction contrast in STEM

In STEM-EELS, the short camera length prevents the usage of the high angle annular dark field detector. Instead, we simply use an annular dark field detector that surrounds the entrance to the spectrometer. At 80 kV, the detector collects electrons that have been scattered through an angle between 55 and 70 mrad. For palladium and palladium hydride, the 440, 600, and 620 reflections fall into the range of collected angles at this voltage. As a result, changes in orientation or phase can cause significant changes in STEM contrast. The spots captured by the detector are shown in Supplementary Fig. 2.

Supplementary References

[1] Niu, W., Zhang, L. & Xu, G. Shape-controlled synthesis of single-crystalline palladium nanocrystals. *ACS Nano* **4**, 1987–1996 (2010).

[2] Bardhan, R. *et al.* Uncovering the intrinsic size dependence of hydriding phase transformations in nanocrystals. *Nat. Mater.* **12**, 905–912 (2013).

[3] Cogswell, D. A. & Bazant, M. Z. Coherency Strain and the Kinetics of Phase Separation in $LiFePO_4$ Nanoparticles. *ACS Nano* **6**, 2215–2225 (2012).
